# Physiotherapy alone or in combination with corticosteroid injection for acute lateral epicondylitis in general practice: A protocol for a randomised, placebo-controlled study

**DOI:** 10.1186/1471-2474-10-152

**Published:** 2009-12-04

**Authors:** Morten Olaussen, Øystein Holmedal, Morten Lindbæk, Søren Brage

**Affiliations:** 1 Grålum Medical Center, Tuneveien 96, NO-1712 Grålum, Norway; 2 Hafslundsøy Medical Center, Helgebyveien 2, NO-1734 Hafslundsøy, Norway; 3 Institute of General Practice and Community Medicine, University of Oslo, PO Box 1130 Blindern, NO-0318 Oslo, Norway

## Abstract

**Background:**

Lateral epicondylitis is a painful condition responsible for loss of function and sick leave for long periods of time. In many countries, the treatment guidelines recommend a wait-and-see policy, reflecting that no conclusions on the best treatment can be drawn from the available research, published studies and meta-analyses.

**Methods/Design:**

Randomized double blind controlled clinical trial in a primary care setting. While earlier trials have either compared corticosteroid injections to physical therapy or to naproxen orally, we will compare the clinical effect of physiotherapy alone or physiotherapy combined with corticosteroid injection in the initial treatment of acute tennis elbow. Patients seeing their general practitioner with lateral elbow pain of recent onset will be randomised to one of three interventions: 1: physiotherapy, corticosteroid injection and naproxen or 2: physiotherapy, placebo injection and naproxen or 3: wait and see treatment with naproxen alone. Treatment and assessments are done by two different doctors, and the contents of the injection is unknown to both the treating doctor and patient. The primary outcome measure is the patient's evaluation of improvement after 6, 12, 26 and 52 weeks. Secondary outcome measures are pain, function and severity of main complaint, pain-free grip strength, maximal grip strength, pressure-pain threshold, the patient's satisfaction with the treatment and duration of sick leave.

**Conclusion:**

This article describes a randomized, double blind, controlled clinical trial with a one year follow up to investigate the effects of adding steroid injections to physiotherapy in acute lateral epicondylitis.

**Trial Registration:**

ClinicalTrials.gov Identifier: NCT00826462

## Background

Lateral epicondylitis of the elbow is characterised by pain and tenderness of the lateral humeral epicondyle and pain on resisted dorsiflexion of the wrist, the 3. digit or both. There is also often pain on resisted radial deviation of the wrist. The condition is a frequent complaint with an overall prevalence of 1-3% [1]. The highest incidence is found in persons 40-60 years old. For women, the incidence increases to 10% between the ages of 42 - 46 [2,3]. The incidence in general practice is estimated to be 4 - 7 per 1000 per year [2,4,5]. The aetiology has been assumed to be over-use damage to the forearm extensor muscles - either minor or non - recognised traumas. There is little evidence of inflammation [6]. Most authors attribute the condition to a lesion in the extensor apparatus at the lateral humeral epicondyle, specifically the short radial extensor muscle [2,7]. Cyriax [8] and Ombregt *et al *[9] identify four subgroups of lateral epicondylitis depending on the exact location of the lesion. In their experience, in 90% of the cases, the lesion is situated in the anterior part of the lateral epicondyle at the origin of the short radial extensor muscle. The second most frequent lesion accounts for 8% of the cases and is localised at the muscle body itself. Lateral epicondylitis usually is a self-limiting condition, but complaints may last up to 2 years or longer [10]. A study from general practice shows that 80% heal within one year on wait-and-see treatment (rest, paracetamol or NSAIDs taken orally) even when initial symptoms had lasted more than 4 weeks [11]. In many countries, treatment guidelines recommend a wait-and-see policy.

Many treatments have been proposed leading to a number of trials, but reviews including several recent meta-analyses have led to no conclusions as to which is the best. This is due to low statistical strength, low internal validity and insufficient study data reporting [12,13]. Schmidt *et al *2003 [14] reviewed literature on physical therapy prior to 1999 and found no evidence of effect, with the exception of ultrasound, where a minor effect was shown. Bisset *et al *2005 [15] published a meta-analysis of 28 randomised studies published before 2003 of different physical therapies for lateral epicondylitis satisfying at least 15 out of 28 criteria (PEDro rating scale)[16]. Most studies had a small number of subjects, and only eight had long term follow-up of effect of therapy. Extra corporeal shock wave therapy was found to have no effect, and manipulation and exercise were found to have only a short-term effect.

A meta-analysis by Smith *et al *2002 [17] on the effect of corticosteroid injections found evidence of short-term pain relief, but no effect beyond the initial 6 weeks. There was however some uncertainty due to few and small studies.

The Cochrane Library has several reviews of treatment for lateral epicondylitis: acupuncture [18], deep transverse friction massage [19], NSAIDS [20], orthosis [21], extra corporeal shock wave therapy [22] and surgery [23]. These reviews all conclude that there is insufficient evidence to draw firm conclusions as to which methods of treatment are effective. However, there are indications that topical NSAIDs and manipulation and exercise have a short term effect. As to NSAIDs taken orally, there is probably a short-term effect, although it is impossible to either recommend use or not. For extracorporeal shockwave therapy, there is evidence to conclude that this treatment has no effect. Ultrasound has a possible short-term effect based on one meta-analysis [14]. In fact, there is scant support for any long-term treatment in the literature.

Looking for a better treatment for epicondylitis, we have found two studies to be of special interest. Both were carried out in primary care settings with one year follow-up. One study compared corticosteroid injection with physical therapy (ultrasound, manipulation and exercise) and a wait-and-see group [11]. The other compared corticosteroid injection with naproxen orally and placebo medication [24]. Both concluded that corticosteroid injection is safe and effective for pain relief during the first 6 weeks, and the effect of this treatment is better than physiotherapy, wait-and-see and naproxen orally within the same time-frame. Smidt *et al *[11] found that physiotherapy gave some, but not significantly better long-term effect than wait-and-see treatment. A more recent study comparing physiotherapy and corticosteroid injection [25] concluded that the significant short term benefits of corticosteroid injection are paradoxically reversed after six weeks, with high recurrence rates and that combining elbow manipulation and exercise has superior benefit to wait and see in the first six weeks and to corticosteroid injections after six weeks. Comparing corticosteroid injection with placebo injection, four clinical trials found no significant effect of corticosteroids at 6 months [26-29] and at 12 months, although one trial reported improvement at 8 weeks [27].

We find there is a good reason to investigate the long-term effects of physiotherapy - this is also recommended in a recent meta-analysis [15]. At the same time, it would be interesting to see whether the good initial response from corticosteroid injection [11,24] may be extended if combined with physiotherapy. We have only found two studies that have evaluated this combination, one found no added effect of corticosteroid injection at 6 months [28], the other had only a 7 week follow up [30]. A protocol for a larger study has recently been published [31].

### Objective

The objective of this study is to compare the clinical effect of physiotherapy alone or physiotherapy combined with corticosteroid injection in the initial treatment of acute lateral epicondylitis in a primary care setting. Also, to find the short and long term effect of physiotherapy and to ascertain whether this outcome is influenced by corticosteroid injection.

## Methods and design

The study is designed as a randomized, placebo-controlled trial in a primary care setting in the city of Sarpsborg and surrounding areas in Ostfold county, Norway. Patients are referred by their general practitioner to one of two trial doctors who make the initial evaluation of inclusion and exclusion criteria, as well as treatment, follow-up and outcome assessments. After a treatment period of six weeks, the patient is followed with assessments for a total of 12 months.

### Participants and recruitment

**Figure 1:**
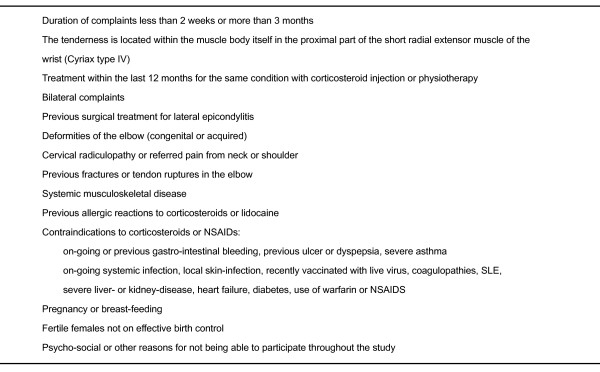
**Exclusion criteria**.

Patients aged 18-70 years seeing their general practitioner with pain of recent onset from the lateral part of the elbow are eligible for inclusion. The other inclusion criteria are pain increase on resisted dorsiflexion of the wrist with the elbow extended and the fingers flexed or pain increase on resisted radial deviation of the wrist or resisted extension of the third finger. We will investigate the acute condition, and will exclude patients with a duration of complaints of more than 3 months, as well as light, self-limiting conditions with a duration of symptoms of less than two weeks. We will exclude patients with tenderness located within the muscle body itself (Cyriax type IV)[8]. Recent treatment with corticosteroid injection or physiotherapy will also exclude the patient. All exclusion criteria are given in figure 1.

### Intervention

In a six-week treatment period, patients will receive one of three treatments:

• Physiotherapy, corticosteroid injection and naproxen

• Physiotherapy, placebo injection and naproxen

• Wait-and-see treatment: naproxen alone

#### Injections

The injections will be blinded to the investigator and given at start and after three weeks, consisting of triamcinolone 10 mg or placebo and lidocaine 10 mg (Triamcinolone: Kenacort-T^® ^"Bristol-Myers Squibb" injection solution 10 mg/ml ATC-no.: H02A B08. Placebo: Sodium Chloride "B. Braun" injection solution 9 mg/ml ATC-no.: V07A B-. Lidocaine: Xylocain^® ^inj. "AstraZeneca" 20 mg/ml ATC-no.: N01B B02). We have chosen to use triamcinolone since it has been used in earlier studies [11,24,25], is readily available and commonly used. One study found significant improvement with triamcinolone compared to lidocaine at 8 weeks and the results are similar with 10 and 20 mg triamcinolone [27]. The injection is given with the patient in a supine position with the elbow flexed and the wrist pronated. The skin is cleaned with alcohol and the most tender point is located. A 0.6 mm needle is inserted at 90 degrees down to the level of bone and then pulled back 1-2 mm. The injection is done by leaving several small depots at the surface of the tendon. After the injection, the patient is tested for pain on resisted dorsiflexion of the wrist and the result recorded. The patient is informed of possible adverse effects from the injection and is advised to avoid pain provoking activities for the rest of the day. After three weeks, possible adverse reactions to the first injection will be recorded. Together with possible increase of pain or symptoms, this is considered before the decision to give a second injection is taken.

#### Physiotherapy

The physiotherapy will be given by two cooperating physiotherapists twice weekly for six weeks. Based on treatments used in earlier studies [11,25] a treatment protocol has been devised comprising treatments that should be well known by most physiotherapists. It will consist of deep transverse friction massage at the tendon origin for 15 minutes, Mill's manipulation [9] once each treatment-session and soft tissue treatment with stretching of the radial wrist extensors. The patients will receive oral and written instructions for home exercises daily for six weeks with eccentric exercise (three times 30 repetitions) and isolated stretching of radial wrist extensors (three times daily for 40 seconds).

#### General treatment and information

All three treatment groups will receive naproxen 500 mg twice daily for two weeks from start (Naprosyn Entero^® ^"Roche" 500 mg. ATC-no.: M01A E02). The reasons for this are mostly pragmatic, since the use of NSAIDs is widespread and the control group thus will receive some form of treatment in the initial six-week period. The treatment is expected to have uniform effect in the three groups. Paracetamol can be taken for pain at the patient's own discretion up to 4 grams daily and use of such extra pain medication will be registered. General advice will be given to all groups, including the natural course of the condition, and expected duration of complaints is discussed. Advice on avoiding pain-provoking use of the elbow is given. The need for sick leave is discussed, but left for the patient's own general practitioner to decide. Additional treatment after the six-week treatment period is given at the discretion of the patient's own general practitioner. There are no restrictions as to what treatment the patient can receive. Additional treatments will be registered on the later assessments by the trial doctors.

### Outcome measures and assessments

The primary outcome measure is the patient's evaluation of improvement after 6, 12, 26 and 52 weeks [11]. Secondary outcome measures include pain, function and severity of main complaint, pain-free grip strength, maximal grip strength, pressure-pain threshold, the patient's satisfaction with the treatment, the need for co-intervention and duration of sick leave.

All assessments are recorded using pre-made, standardised forms. Outcome assessments are done by the trial doctors and by the patient answering a questionnaire. Based on earlier studies and assessment of the validity and reliability of the outcome measures, the patient's evaluation of improvement will be registered on a 6-point Likert scale (much worse - worse - a little worse - some improvement - much improvement - completely recovered) [11]. Elbow pain, to what extent the use of the elbow is affected and severity of main complaint is registered on a Visual Analogue Scale [11,24,32]. Pain-free grip strength and maximum grip strength will be registered with a hand held, analogue dynamometer as a mean of three measurements in a ratio of affected to unaffected side (Jamar Hydraulic Hand Dynamometer - 5030J) [32,33]. Pressure-pain threshold over the epicondyle will be measured with an analogue algometer, also as a mean of three measurements in a ratio of affected to unaffected side. (Wagner Instruments, Algometer FPK 20) [32,33]. Pain on resisted dorsiflexion will be registered on a 3-point scale (none, some, definite)[24]. Whether the patient experiences pain on eight every-day activities (dressing, eating, washing, household tasks, opening doors, carrying objects, with work, at sports) will be registered using a pain-free function questionnaire [11,24].

**Figure 2:**
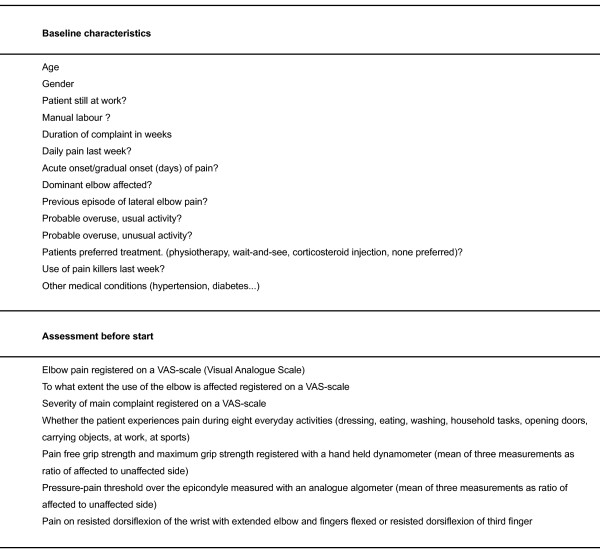
**Baseline characteristics and assessment before start**.

**Figure 3:**
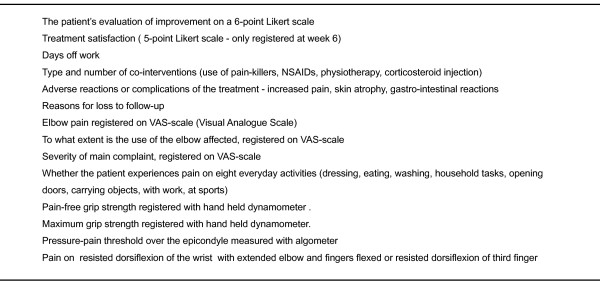
**Outcome measures after 6, 12, 26 and 52 weeks**.

Before start, a number of baseline characteristics will be registered and an assessment will be done (figure 2). After 6, 12, 26 and 52 weeks, the following assessments will be done: the patient's evaluation of improvement on a 6-point Likert scale, treatment satisfaction (done only after 6 weeks on a 5-point Likert scale), days off work, type and number of co-interventions (use of pain-killers, NSAIDs, physiotherapy or corticosteroid injection), adverse reactions or complications of the treatment (increased pain, skin atrophy, gastro-intestinal reactions) and reasons for loss to follow-up. In addition, the assessments done before start will be repeated, registering elbow pain, to what extent the use of the elbow is affected and the severity of main complaint, whether the patient experiences pain during eight everyday activities, pain-free grip strength and maximum grip strength, pressure-pain threshold and pain on resisted dorsiflexion of the wrist (figure 3).

**Figure 4:**
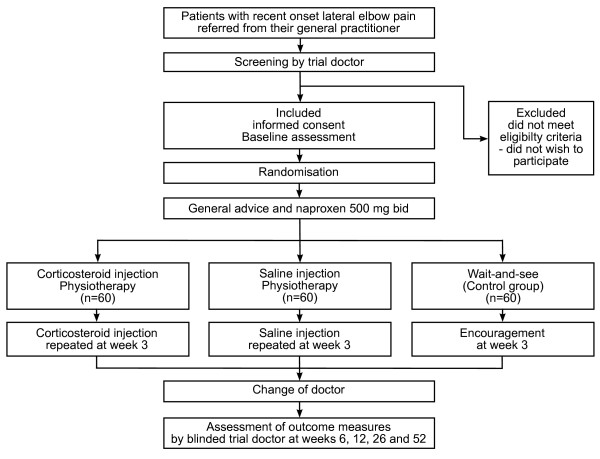
**Overview of the study design**. An overview of the study design showing recruitment, assessments, the three treatment groups, follow-up and outcome assessments.

The patient is also seen by the trial doctor at 3 weeks for a possible second injection if in the appropriate group. Compliance to the naproxen treatment will be monitored by counting remaining tablets. An overview of the study design is given in figure 4.

### Randomisation and blinding

A computerised randomisation schedule will be prepared by an independent researcher, and using concealed allocation each patient will be assigned to one of three treatments using numeric block randomisation. The patient will first see one of the trial doctors, who is responsible for inclusion, baseline assessment and treatment. This doctor will not know the content of the injections, since these will be prepared by a research assistant. The contents of the syringe are covered by an opaque adhesive patch to further disguise it. This way, the injections can be made double blind for the doctor and patient. From week six, the patient will see the other trial doctor, who will be unaware of which treatment the patient has received, and patients will be cautioned at each assessment about not disclosing what kind of initial treatment they received. The assessments will thus be performed blinded. The success of blinding will be assessed at 52 weeks by the trial doctor guessing which treatment the patient received.

### Sample size and statistical analysis

Sample size is based on the ability to detect a 25% difference in the success rate (defined as "much better" or "completely recovered" on a 6-point Likert-scale) between the groups at three months. Prior to three months, earlier studies show a very large success rate on treatment with corticosteroid injections[11,25]. Later in the course of the condition, the success rate will increase regardless of intervention. Based on earlier studies [11], we have assumed a success rate of 55% at three months in the least successful group. The target sample size is estimated at 52 patients per group (two tailed α: 0.05, β: 0.20) giving a total of 156 patients. Assuming a loss-to-follow-up of 10% and practicalities in allocating the patients to 3 groups, we will include 180 patients in three treatment groups of 60 patients. With an incidence of 4-7/1000 per year and 50.000 people living in the Sarpsborg area, we estimate a period of inclusion of 1 to 1.5 years. The statistical analysis will be based on intention-to-treat analysis.

### Ethics and Data Security

The trial has been approved by The Regional Committee for Research Ethics in Norway, The Norwegian Social Science Data Services and The Norwegian Medicines Agency, and all patients will be asked for written informed consent on standardised forms. All patient data will be de-identified before use in research. The data will be kept de-identified on forms and in computers inaccessible to unauthorised persons. The list linking each patient's name and personal data will be kept separate, accessible only to the two trial doctors. In compliance with government regulations, personal data will be kept de-identified for a further period of 15 years after the end of the trial before deletion.

### Adverse reactions

Adverse reactions or side effects to naproxen, triamcinolone or physiotherapy would be grounds for dismissing the patient from the trial. These factors will be continuously appraised during the trial. Side effects to the treatment with drugs will be reported in compliance with local Norwegian law.

## Discussion

Earlier studies, including meta-analyses, have concluded that more research on treatment options for lateral epicondylitis is needed. Evidence based treatments guidelines will help in making effective and sound treatment decisions. This study is designed to mirror the normal work flow in a primary care setting, making the results readily applicable in this setting. We have selected recent-onset complaints as opposed to recurring or chronic forms for easier comparison with earlier studies, and to make the results more applicable in general practice, where most patients with acute lateral epicondylitis are seen. As to physiotherapy, we are cooperating with two clinical physiotherapists engaged in normal practice, and have developed a treatment protocol reflecting usual treatments for this condition. By assessing the patients' time off paid employment, we also hope to gain valuable knowledge about the economic impacts of this condition. To address the methodological problems in some earlier research, we have designed our trial as a double blind, randomised controlled study with a one year follow up. An effort has been made to blind the injections to both investigator and participant for more valid results. We have calculated sample size and statistical strength as shown above to produce valid results. The study will comply with the CONSORT Statement [34] and the study protocol is published, enabling later comparison of what was originally intended with what was actually done, thus preventing both "data dredging" and post-hoc revisions of study aims.

## Competing interests

The authors declare that they have no competing interests.

## Authors' contributions

MO conceived the trial. MO and ØH prepared the trial, wrote the manuscript and are the chief investigators. ML and SB supervised the planning and participated in the design of the trial. MO, ØH, ML and SB edited and revisedthe manuscript. All authors have read and approved the final manuscript.

## Pre-publication history

The pre-publication history for this paper can be accessed here:

http://www.biomedcentral.com/1471-2474/10/152/prepub
